# Supplementary material for: Breast cancer incidence in Yogyakarta, Indonesia from 2008–2019: A cross-sectional study using trend analysis and geographical information system
Source: PLoS One. 2023 Jul 5;18(7):e0288073. doi: 10.1371/journal.pone.0288073 (PMC10321628; doi:10.1371/journal.pone.0288073)
Supplement: S1 Table — (PDF) [file pone.0288073.s001.pdf]

S1 Table. Age-standardized rate of breast cancer among subdistricts level in the three catchment districts of Yogyakarta Province (2008-2019)

District	Subdistrict	N	ASR	Standard Error
Sleman	Berbah	76	32.76	3.76
Sleman	Cangkringan	45	32.95	4.91
Sleman	Depok	336	62.70	3.42
Sleman	Gamping	190	48.80	3.54
Sleman	Godean	137	45.63	3.90
Sleman	Kalasan	108	32.51	3.13
Sleman	Minggir	53	33.65	4.62
Sleman	Mlati	164	42.69	3.33
Sleman	Moyudan	74	48.47	5.63
Sleman	Ngaglik	187	45.95	3.36
Sleman	Ngemplak	73	28.65	3.35
Sleman	Pakem	62	38.05	4.83
Sleman	Prambanan	70	30.70	3.67
Sleman	Seyegan	81	37.58	4.17
Sleman	Sleman	131	45.36	3.96
Sleman	Tempel	67	28.40	3.47
Sleman	Turi	60	38.70	5.00
Yogyakarta City	Danurejan	61	65.99	1.74
Yogyakarta City	Gedongtengen	40	42.95	8.45
Yogyakarta City	Gondokusuman	98	52.33	6.79
Yogyakarta City	Gondomanan	41	59.62	5.29
Yogyakarta City	Jetis Kota	54	43.85	9.31
Yogyakarta City	Kotagede	60	43.41	5.97
Yogyakarta City	Kraton	69	68.34	5.60
Yogyakarta City	Mantrijeron	96	62.45	8.22
Yogyakarta City	Mergangsan	84	58.91	6.37
Yogyakarta City	Ngampilan	45	54.03	6.43
Yogyakarta City	Pakualaman	35	70.38	8.05

Yogyakarta City	Tegalrejo	69	43.60	11.89
Yogyakarta City	Umbulharjo	175	61.38	5.25
Yogyakarta City	Wirobrajan	52	43.62	4.64
Bantul	Bambanglipuro	78	42.81	4.85
Bantul	Banguntapan	171	38.62	2.95
Bantul	Bantul	145	52.88	4.39
Bantul	Dlingo	26	15.35	3.01
Bantul	Imogiri	68	25.16	3.05
Bantul	Jetis Bantul	79	31.69	3.57
Bantul	Kasihan	163	39.26	3.07
Bantul	Kretek	39	27.57	4.41
Bantul	Pajangan	53	36.84	5.06
Bantul	Pandak	78	34.85	3.95
Bantul	Piyungan	55	26.26	3.54
Bantul	Pleret	41	21.65	3.38
Bantul	Pundong	48	31.09	4.49
Bantul	Sanden	35	23.64	4.00
Bantul	Sedayu	66	33.82	4.16
Bantul	Sewon	168	41.10	3.17
Bantul	Srandakan	62	44.73	5.68

Abbreviation: ASR: Age-standardized rate
