# Supplementary material for: Breast cancer incidence in Yogyakarta, Indonesia from 2008–2019: A cross-sectional study using trend analysis and geographical information system
Source: PLoS One. 2023 Jul 5;18(7):e0288073. doi: 10.1371/journal.pone.0288073 (PMC10321628; doi:10.1371/journal.pone.0288073)
Supplement: S2 Table — (PDF) [file pone.0288073.s002.pdf]

S2 Table. Breast cancer data quality in Yogyakarta PBCR through data collection period (2016-2022)

Data collection period	N	Data quality evaluation	
		%MV	%DCO
2016-2017	664	64.46	0.15
2016-2018	1,134	71.16	0.09
2016-2019	1,887	72.02	0.11
2016-2020	2,679	72.64	0.15
2016-2021	3,797	72.32	0.13
2016-2022	4,268	73.81	0.12

Abbreviation: PBCR: Population-based cancer registry; %MV: Morphology verified; %DCO: Death certificate only
